# Proteid Dietaries in Nephritis

**Published:** 1909-05-15

**Authors:** 


### Medicine.

### PROTEID DIETARIES IN NEPHRITIS.

There are probably as great differences of opinion upon the question of what proteids, and what quantities of them, are permissible in cases of nephritis as upon any other in medicine. The following table, taken from a paper by Professor Gouget in the *Gazette des Hôpitaux*, shows the richness in albuminoids, fats, and carbohydrates of several foodstuffs, and it serves as a guide to the question under discussion:—

| | Proteid in parts per 1,000 | Fat in parts per 1,000 | Carbohydrate in parts per 1,000 |
|---|---|---|---|
| Hen's egg | 132 | 119 | 4 |
| Beef | 210 | 54 | 5 |
| Ham | 160 | 346 | 5 |
| Sweetbread | 220 | 4 | 0 |
| Calves' kidneys | 221 | 28 | 0 |
| Brains | 105 | 153 | 11 |
| Salmon | 216 | 127 | 0 |
| Eels | 128 | 283 | 0 |
| Potatoes | 16 | 1 | 200 |
| Manioc | 12 | 4 | 283 |
| Chestnuts | 57 | 16 | 384 |
| Oatmeal | 110 | 49 | 674 |
| Barleymeal | 105 | 17 | 696 |
| Wheatflour | 102 | 10 | 737 |
| Bread | 70 | 7 | 538 |
| Peas | 220 | 16 | 538 |
| Lentils | 238 | 18 | 528 |
| Gruyère cheese | 300 | 284 | 21 |
| Brie (cream cheese) | 179 | 253 | 35 |
| Camembert (cream cheese) | 179 | 253 | 35 |
| Spinach | 35 | 6 | 44 |
| Carrots | 12 | 3 | 92 |
| Chocolate | 62 | 210 | 676 |

Apart from the quantity of proteid food, does the kind of proteid matter? Are the albuminoids of milk or of vegetables better borne than those of eggs or of fish, or of meat? Claude Bernard and Stokvis having succeeded in producing albuminuria by the exhibition of a certain number of raw eggs, there has been a tendency to regard egg-albumin, which is relatively rich in sulphur, as particularly harmful, and some observers, such as Semmola, proscribe eggs in nephritis cases altogether. As a matter of fact, however, even though it might be unwise to give raw eggs in these cases, cooked eggs in moderate amount, and particularly their yolks, are by no means contra-indicated, and some authorities prefer them to meat. The latter is regarded by Germain See and others as the worst possible food in all cases of nephritis, yet some allow white meats, and not the red, which were formerly thought to be the richer in extractives. We know now, however, thanks particularly to the researches of Von Noorden and his pupils, that there is really no reason for preferring the former to the latter. At the same time, experience has shown that certain meats, such as fresh pork and ham that has not been salted, are particularly well borne by renal cases; whereas veal, especially very young veal, which gives rise to an eminently fermentible gelatine in the bowel, is far less good. According to Robin, beef should be better than mutton, or even chicken. As regards fish, regarded with favour by Klemperer, excluded by Teissier and Renaut, it is probably neither better nor worse than meat when fresh. Lastly, vegetable proteids would seem to offer no decided superiority over animal nitrogenous compounds unless perhaps in so far as a greater percentage of them remains undigested—3 per cent. to 15 per cent.—which allows of a larger bulk being permitted without overstepping the nitrogen limits that may have been laid down in any particular case.

In acute uræmia the dietary must necessarily be very restricted, consisting for the most part of water only, with or without the addition of lactose. This, however, can scarcely be persisted with for more than eight-and-forty hours. If at the end of that time milk has to be substituted for water, and if this is not well borne, it may be necessary to replace it partly by some of the farinaceous substances mentioned below.

The milk or milk and farinaceous *régime* is indicated in quite acute nephritis; in the acute exacerbations of chronic nephritis; in cases in which uræmia threatens or is actually present; and in Bright's disease with severe cardiac failure. It is also wise to adopt it, initially and tentatively at any rate, in cases of subacute nephritis with much œdema, and in general in all cases of nephritis whose previous evolution is unknown; it tends above all other measures to increase the quantity of urine passed, and it is therefore to be employed in all renal conditions in which the urine, hitherto normal or increased in amount, is diminishing.

A milk dietary should not be persisted with too slavishly however. It should be adopted provisionally under the above circumstances, but if the patient ceases to make progress, and especially if no increase in the volume of urine passed is brought about, the quantity of milk given may be decreased and a corresponding amount of rice, arrowroot, sago, tapioca, chestnuts, or carrots allowed—cooked in some palatable and pleasant form. Potatoes, which contain much water and relatively far more carbohydrates than albuminoids, and which have so abundant a residue that the tendency to constipation is materially lessened, have been particularly advocated by some; cooked greens, ripe grapes, stewed fruits, and fruit jellies being also allowed. A little later, if these are tolerated well, various foods made from wheat flour may be ordered, such as bread, toast, or biscuits. Then will come fresh butter, the yolks of eggs, and puddings; next, but only at the mid-day meal, the products of peas, beans, or lentils, the effects of which have to be very carefully watched, because foods prepared from them are apt to be ill-digested owing to their richness in proteids and salts; eventually, one reaches cooked eggs, fresh fish, fresh pork or ham, and at last other meats that have been well boiled, stewed, or roasted without too much gravy or sauce. Such are the various steps that are usually taken in succession, more or less quickly according to the case. Sometimes it is impossible to pass on further than the lacto-vegetarian phase.

Another aspect of the diet which may be of extreme importance is its richness in sodium chloride and its relative proportions of salt and of nitrogen. Roughly speaking, it is most important to curtail the sodium chloride chiefly in cases of parenchymatous nephritis with œdema, and to limit the nitrogen particularly in patients with granular kidneys. At the same time it should be remembered that this holds good only in the most general way, and that the chlorides have to be watched in granular kidney cases and the nitrogen in acute nephritis. There can be little doubt that a milk diet in scarlatina patients, begun early and continued with for several weeks, is one of the surest means of preventing the nephritis of convalescence. It has been shown, however, that a much more generous dietary in which the chloride of sodium is alone restricted achieves the same end, with this advantage, that the better-fed patients convalesce more rapidly.

